# What’s needed to achieve zero leprosy

**DOI:** 10.2471/BLT.24.292037

**Published:** 2024-08-01

**Authors:** Yohei Sasakawa

**Affiliations:** a The Nippon Foundation, The Nippon Zaidan Building 1-2-2 Akasaka, Minato-ku Tokyo 107-8404, Japan.

Leprosy, or Hansen’s disease, is now curable, and the number of cases has fallen dramatically, especially since the introduction of the World Health Organization (WHO)-recommended multidrug therapy regimens in the 1980s. In 2022, 174 087 new cases were reported,^1^ compared to an estimated global prevalence of 12 million patients in 1985.^2^

I like to describe the approach to leprosy in terms of the two wheels of a motorcycle: the front wheel represents measures to diagnose and treat the disease; the rear wheel represents initiatives to eliminate stigma and discrimination. If the two wheels do not turn together, we will not make progress.

A few decades ago, the fight against leprosy attracted political support. The 44th World Health Assembly in 1991 adopted a resolution to eliminate leprosy as a public health problem by the year 2000,^3^ defining elimination as a prevalence of less than one case per 10 000 population at the global level. Governments became actively involved and the goal was achieved. By 2010, this milestone had also been attained at the national level in almost all endemic countries except Brazil and some island countries with a population of less than 1 million.

Progress has also been made in addressing stigma and discrimination. In December 2010, at the 65th session of the United Nations (UN) General Assembly, a resolution on the elimination of discrimination against persons affected by leprosy and their family members^4^ was adopted by all 192 Member States at the time, together with accompanying principles and guidelines.^5^ As a result, the international community has come to see leprosy as a human rights issue.

The following year, WHO developed guidelines to strengthen the participation of persons affected by leprosy in services including counselling, disability prevention, rehabilitation and research.^6^ Meanwhile, organizations of people affected by leprosy are becoming more organized, active and networked. Currently, such organizations are known to exist in 32 countries.^7^

Despite this progress, leprosy is unfortunately still present. On the medical front, budgets and staffing have been drastically reduced since leprosy was eliminated as a public health problem. As a result, health ministries have not actively pursued early detection activities, resulting in hidden cases and current statistics that do not always reflect the true numbers.

On the social front, governments have not fully implemented the principles and guidelines adopted by the UN because they are not legally binding. Consequently, discrimination against persons affected by leprosy remains deeply entrenched in many parts of the world. For example, 139 discriminatory laws relating to leprosy still exist in 24 countries.^8^

In 2021, WHO released *Towards zero leprosy: global leprosy (Hansen’s disease) strategy 2021–2030*.^9^ The strategy’s targets include a 70% reduction in the annual number of new cases detected; a 90% reduction in the rate per million population of new cases with grade-2 (visible) disability; 120 countries reporting zero new autochthonous cases (up from 45 countries in 2019); and the amendment or elimination of all remaining discriminatory laws.

Furthermore, in 2023, WHO set new milestones and indicators for leprosy as part of a new Leprosy Elimination Framework. First, interruption of transmission, defined as no new autochthonous cases among children for five consecutive years. Second, in the next phase, elimination of disease, defined as no new autochthonous cases for three consecutive years and no child cases in five years.^10^

To achieve these targets, WHO is promoting new interventions such as leprosy post-exposure prophylaxis with single-dose rifampicin, and the integration of leprosy into other neglected tropical diseases programmes.

What I want to emphasize here is how to achieve these goals. Based on my experience of visiting over 100 countries as WHO Goodwill Ambassador for Leprosy Elimination, the following are key: an expression of political will at the leadership level in each country, budgetary commitments, and securing and allocating the necessary human resources.

I believe we have reached the last mile of our 100-mile journey against leprosy. But this last mile is the most difficult. In February 2024, at the age of 85, I climbed the highest mountain in Africa, Mount Kilimanjaro, as part of a campaign to spread the message “Don’t Forget Leprosy” around the world – a campaign that received the support of global leaders such as Pope Francis and WHO Director-General Tedros Adhanom Ghebreyesus. The final ascent was a challenge, but I made it. Following this experience, let me add one more key to completing the last mile: a never-give-up spirit.
